# RPL22 Overexpression Promotes Psoriasis-Like Lesion by Inducing Keratinocytes Abnormal Biological Behavior

**DOI:** 10.3389/fimmu.2021.699900

**Published:** 2021-06-18

**Authors:** Jinrong Zeng, Yue Zhang, Hanyi Zhang, Yuezhong Zhang, Lihua Gao, Xiaoliang Tong, Yajie Xie, Qian Hu, Chunli Chen, Shu Ding, Jianyun Lu

**Affiliations:** ^1^ Department of Dermatology, Third Xiangya Hospital, Central South University, Changsha, China; ^2^ XiangYa School of Medicine, Central South University, Changsha, China

**Keywords:** psoriasis, RPL22, H3K27ac, CXCL10, CyclinD1, keratinocytes

## Abstract

**Background:**

Keratinocytes of psoriasis have anti-apoptotic properties including delayed apoptosis process, accelerated proliferation metabolism and postponed differentiation process. However, the specific mechanism leading to the abnormal biological behavior of keratinocytes remains unclear.

**Objectives:**

We investigated the role of increased RPL22 expression in regulating the abnormal biological behavior of keratinocytes and the mechanism of regulation of RPL22 expression in skin lesions of psoriatic patients.

**Methods:**

We examined clinical samples and utilized cytokine-induced cell and IMQ-treated mouse models. We determined the expression and functions of RPL22 *in vitro* and *in vivo*.

**Results:**

We showed that RPL22 expression was significantly increased in the skin lesions of psoriasis patients and IMQ-treated psoriatic-like mice. Such increased expression is attributed to hyperacetylation of histone H3K27 in the promoter region of RPL22. Interestingly, overexpression of RPL22 enhanced keratinocyte proliferation by increasing cyclinD1 expression and accelerated CD4^+^T cells recruitment *via* upregulating CXCL10 expression. Finally, we demonstrated that RPL22 overexpression promoted psoriasiform phenotypes in IMQ-induced mouse skins.

**Conclusions:**

These findings suggested that RPL22 regulates keratinocytes abnormal biological behavior and contributes to the development of psoriatic phenotypes. Thus, RPL22 might be a novel potential molecular target for treatment of psoriasis.

## Introduction

Psoriasis is a common and relapsing chronic inflammatory skin disease which is mainly characterized by excessive proliferation of keratinocytes (KCs) and skin inflammation (1). KCs are not only effector cells, but also special antigen-presenting cells, which produce antimicrobial peptides and cytokines and thus play an important role in the pathogenesis of psoriasis (2, 3). When exposed to environmental factors, keratinocytes can activate T cells by presenting LL37, CD1a, ADAMTSL5, viral or bacterial products (4–6). Ulteriorly, T cells interact with KCs and promote the release of antimicrobial peptides (LL-37, β-defensins and S100A7), chemokines (CXCL1, CXCL8, CCL20) and cytokines (IL-1β, IL-6, IL-23) (7–9) in KCs. The production of proinflammatory factors by KCs further enhances the recruitment of leukocytes and consequently amplify inflammatory cascade. In addition, KCs-derived growth factors such as platelet-derived growth factor (PDGF) and vascular endothelial growth factor (VEGF) can affect the proliferation of supportive stromal cells. Excessive production of various growth-promoting factors by activated stromal cells, such as keratinocyte growth factor (KGF), can induce KCs hyperproliferation (9). Numerous studies have shown that KCs in psoriatic lesions have anti-apoptotic properties which include delaying apoptotic process and accelerating proliferative metabolism. As the KCs differentiation process is delayed, the epidermal turnover time is shortened from normal 28 days to 3-4 days, eventually showing pathological changes of hyperkeratosis, dyskeratosis, disappearance of granular layer and acanthosis (9, 10). However, the specific mechanisms involved in the regulation of abnormal proliferation and apoptosis of KCs remain unclear.

Ribosomal protein L22 (RPL22), a 128-amino acid RNA-binding protein, is a component of the large 60S ribosomal subunit. RPL22 is not required for protein synthesis (11, 12). Recent studies show that RPL22 can control tumor cell proliferation and migration *via* various signal pathways such as activating NF-κB, Lin28B and p53 (13, 14). However, the aberrant expression and upstream regulatory mechanisms of the RPL22 gene in psoriatic lesions, as well as the importance in the pathogenesis of psoriasis, have not been reported.

In our study, we validated that RPL22 expression was significantly upregulated in psoriatic skin lesions and hyperacetylation of histone H3K27 in the promoter region of RPL22 contributed to its increased expression. Overexpression of RPL22 (i) regulates cell cycle by increasing cyclinD1 expression to promote keratinocyte proliferation and inhibit apoptosis; as well as (ii) accelerate CD4^+^T cells recruitment *via* inducing CXCL10 expression. In IMQ induced psoriasis-like mouse skins, RPL22 overexpression accelerating the development of psoriasis. These findings suggested that RPL22 regulated keratinocytes abnormal biological behavior to involve in the occurrence and development of psoriasis which provided a novel potential molecular target for treatment of psoriasis.

## Materials and Methods

### Patients and Samples

The recruited patients (n=23) who have been initially diagnosed as psoriasis vulgar (PV) by skin histopathological examination in dermatological clinics were included in this study, and the age- and sex- matched healthy individuals (n=23) were recruited from staff at the Third Xiangya Hospital of Central South University. Disease severity was assessed by psoriasis area and severity index(PASI) score. This study was approved by the Ethics Committee of the Third Xiangya Hospital of Central South University. All subjects were informed and signed consent prior to participation in the study.

### Mice

The Balb/c mice were purchased from Slack company (Shanghai, China). 6-8-week-oldfemale mice were used for all experiments. All animals were handled and housed in Animal Experiment Center of Central South University facilities and all operations were in accordance with National Institutes of Health Animal Care and Use Committee guidelines. The animal ethics approval number is 2020-S162.

### Cell Culture

The KCs cell line HaCaT was obtained from China Center for Type Culture Collection (cat no.GDC0106; Wuhan, China) and were cultured in DMEM (Gibco, Thermo Fisher Scientific, Inc.) supplemented with 10% fetal bovine serum (FBS; Gibco, Thermo Fisher Scientific, Inc.) at 37°C with 5% CO_2_. Human primary keratinocytes were obtained from foreskin tissues which were cut into 2mm*1mm pieces and were digested with epidermal-dermal separation solution for overnight at 4°C. 4 ml of epidermal cell separation solution was added for a 15-min digestion which was terminated with serum-containing DMEM medium.

### Pro-Inflammatory Cytokines-Induced Psoriasis-Like Cell Model

The psoriasis-like cell model was established as previously described (15, 16). HaCaT cells were seeded in cell plates at appropriate densities, and after 12 hours of culture, the cells were stimulated with 10 ng/ml M5, a cocktail of inflammatory cytokines including IL-17A (cat no.200-17, Peprotech, USA), IL-22 (cat no.200-22, Peprotech, USA), IL-1α (cat no.200-01A, Peprotech, USA), oncostatin M (cat no.300-10H, Peprotech, USA), and TNF-α (cat no.300-01A, Peprotech, USA) for 24-48 hours before collection for the next experiment. The control group was treated with PBS.

### RNA Isolation and RT-qPCR

Total RNA was isolated from skin or cells by TRIzol^®^ reagent (Invitrogen; Thermo Fisher Scientific, Inc.) according to manufacturer’s guidelines. First‐strand cDNA was synthesized from 800ng total RNA using the PrimeScript^®^ RT reagent kit (TaKaRa, Japan). The qPCR was carried out on a LightCycler^®^96 (Roche Diagnostics, Basel, Switzerland) thermocycler and RNA was measured by SYBR Premix Ex Taq II (TaKaRa, Japan). 2^-(Ct gene-Ct GAPDH)^ and 2^-(∆Ct experiment group-∆Ct control group)^ were used for relative gene expression and fold change of gene expression calculation.

### Western Blot

Total-protein extraction was performed using RIPA buffer containing protease inhibitors (Beyotime, China). After total protein separation and membrane transfer (Millipore, USA), the membranes were immunoblotted with specific primary antibodies, rabbit anti-RPL22(1:1000; Abcam, catalog ab229458), mouse anti−GAPDH (1:5,000; cat no. 60004-1-Ig; Proteintech, USA), at 4°C overnight. Corresponding (ab195380, 1:1000; Abcam) HRP-conjugated affinipure goat anti-rabbit IgG(H+L) (1:5,000; cat no. SA00001-2; Proteintech, USA) and goat anti-mouse IgG(H+L) (1:5,000; cat no. SA00001-1; Proteintech, USA) secondary antibodies were incubated at room temperature for 2 h. The data was analyzed using a GE−ImageQuant LAS 4000 mini (GE Healthcare). The images were cropped for presentation.

### Small Interfering RNA and Plasmid Transfection of Cells

Primary human KCs and HaCaT cells were transfected according to the manufacturer’s instructions using riboFECT CP Transfection Kit (RiboBio, China) or Lipofectamine^®^ 2000 (Invitrogen, USA) with RPL22 siRNA (si-RPL22), RPL22 overexpression plasmid (OE-RPL22) or their corresponding controls. si-RPL22 and negative control (si-NC) were obtained from Guangzhou RiboBio Co. (Guangzhou, China), OE-RPL22 and negative control (OE-NC) was provided by GenScript Co. (USA).

### Cell Apoptosis Assay

Transfected primary KCs were cultured for 48 hours. Cells were collected and then detected with a Fluorescein Isothiocyanate Annexin V Apoptosis Detection kit II (BD Pharmingen; BD Biosciences, Franklin Lakes, NJ, USA) according to the manufacturer’s protocol. The percentage of apoptotic cells were measured by using a flow cytometer (BD Canto II; BD Biosciences). The data were analyzed by FlowJo version 10 (FlowJo LLC, Ashland, OR, USA).

### Cell Proliferation Assays

Transfected HaCaT cells were cultured for 24h, 48h, or 72h. Cell viability was determined by Cell Counting Kit-8 (CCK-8) kit (Dojindo, Kumamoto, Japan). 100 μl fresh media containing 10 μl CCK-8 was added in each well and then the plate was incubated for 2h at 37°C. The cell viability was detected at 450 nm using an EnSpire Multimode Plate Reader (PerkinElmer, Inc., Waltham, MA, USA).

### Cell Cycle Assay

Transfected HaCaT cells were cultured separately for 48h. The cells were collected and fixed with 70% ethanol in cold ice, then washed with PBS, and then stained with PI at 4°C. Finally the cell cycle distribution was detected by flow cytometry.

### Total CD4^+^T Cells Isolation

Peripheral blood mononuclear cells (PBMCs) were separated from the peripheral blood of psoriasis patients by density gradient centrifugation (GE Healthcare, Switzerland). CD4^+^T cells were isolated by positive selection using Miltenyi beads according to the manufacturer’s instructions (Miltenyi, Germany) in sterile conditions. Then, the cells were cultured or collected for subsequent experiments.

### Chemotaxis Assay

When the confluence of transfected KCs reached 80%, the cells were transferred into the bottom chamber of transwell. The extracted CD4^+^T cells were dilated into 2 × 10^6^ cell/mL and added to the upper chamber of transwell. The transwells system was incubated for 90 min at 37°C containing 5% CO_2_ for migration. The migrated T cell populations were analyzed using a florescence activated cell sorter (FACS) machine (Becton Dickinson, Oxford, UK).

### Immunohistochemical Staining

Skin tissues were fixed in formalin for overnight at room temperature and embedded in paraffin. After deparaffining and antigen retrieval, the tissues were incubated for 1h at 37°C with the primary antibodies, namely rabbit anti-RPL22(1:1000; Abcam, catalog ab229458). The samples were washed and incubated for 20min with appropriate conjugated secondary antibodies and Opal 690 Fluorophore (NEL821001KT, PerkinElmer, USA) respectively. After washed by PBST, the slides were counterstained with DAPI. Image analysis was performed using a fluorescent microscope DMI 4000B (Leica, Germany) and Leica Qwin Std analysis software.

### ChIP-qPCR

The skin samples were obtained from 3 psoriasis patients and 3 healthy people respectively. Cell membrane was dissolved with a detergent to release the cell components. The chromatin DNA was then sheared to an average fragment length of 200–1000 bp using a Branson 450 Digital Sonifier Branson, USA. The sheared DNA was diluted and transferred to a strip well for binding with chip-grade anti-acetylated-H3K27 (Abcam, catalogab4729) or anti-normal rabbit IgG negative control and incubated at room temperature for 60 min according to the manufacturer’s instructions of SimpleChIP^®^ Enzymatic Chromatin IP Kit(CST, #9003, USA). Protein was removed by proteinase K and the DNA was quantified by qPCR.

### IMQ-Induced Psoriasis-Like Mouse Model and Treatment

Female Balb/c mouse (6-8 weeks of age) were kept under proper conditions. The mouse were treated with a daily topical dose of 62.5 mg of IMQ cream (5%) (Sichuan Med-shine Pharmaceutical, H20030128, China) on the shaved back for 6 consecutive days. Control mice were treated with the same dose of vehicle cream. All procedures were approved and supervised by the Medicine Animal Care and Use Committee of the Third Xiangya Hospital of Central South University. Female BALB/c mice (6~8 weeks of age) were injected intradermally with si-RPL22 (2.5nmol in 150 μl PBS) or OE-RPL22 (100ug in 150 μl PBS) as well as their controls, on days 1, 2, and 3 during the application of IMQ, si-RPL22 and si-NC were obtained from Guangzhou RiboBio Co. (Guangzhou, China). OE-RPL22 and OE-NC was provided by GenScript Co. (USA). All mice were photographed daily to evaluate the phenotypic characteristics. On the 6th day, the mice that were about to be executed were placed in a transparent airtight device which were filled with a certain amount of diethyl ether, and observed until they died and collected their skin lesions.

### Histological Analysis

The skin tissue was fixed in formalin and embedded in paraffin(Servicebio China). Sections (6 mm) were stained with hematoxylin and eosin (H&E) and stored at room temperature. Epidermal hyperplasia (acanthosis) and the number of dermal infiltrating cells were assessed as histological features. For measuring acanthosis, the epidermal area was outlined, and its pixel size was measured using the lasso tool in Adobe Photoshop CS4. The relative area of the epidermis was calculated using the following formula: area=pixels/(horizontal resolution×vertical resolution).

### Statistical Analyses

All diagrams and graphs analysis was conducted using GraphPad Prism 6.0 (GraphPad Software, Inc.) and the data were presented as the average value± SEM. We assessed data for normal distribution and similar variance between groups. Student’s t-test was used to compare the mean of two independent samples with normal distributions. Correlation and partial correlation analyses were performed by using Pearson’s *r* test. P<0.05 was considered to indicate a statistically significant difference.

## Results

### RPL22 Expression Is Elevated in Skin Lesions of Psoriasis Patients and IMQ-Treated Mice

**Figure 1:**
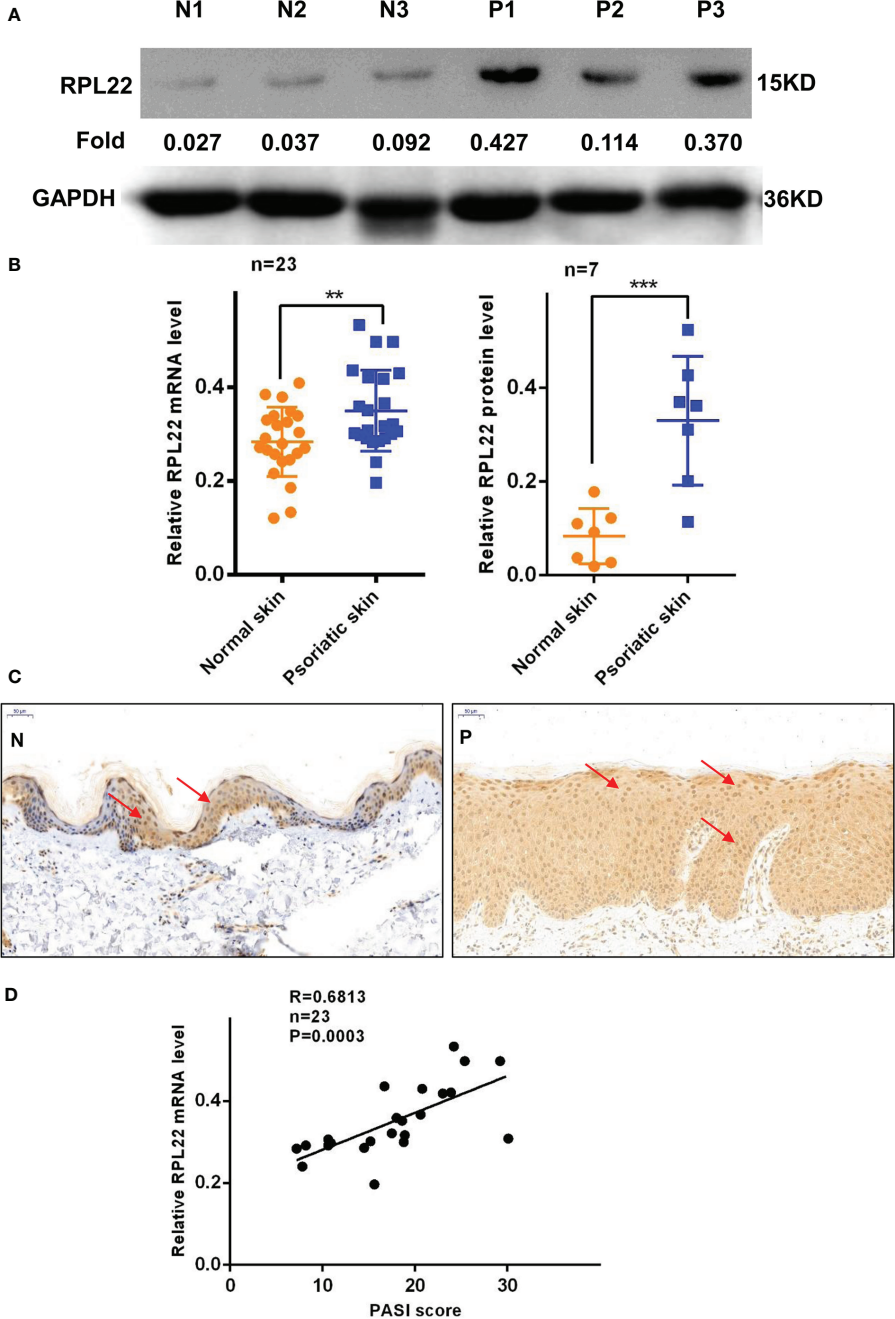
RPL22 expression is elevated in skin lesions of psoriasis patients. **(A)** The protein expression level of RPL22 in skin lesions derived from psoriasis patients and healthy controls. **(B)** The statistical analysis of the relative mRNA (left panel, n = 23) and protein expression levels(right panel, n = 7) of RPL22 in skin lesions derived from psoriasis patients and healthy controls. **(C)** IHC staining of RPL22 in skin lesions derived from psoriasis patients and healthy controls. **(D)** Correlation of human RPL22 mRNA expression in psoriatic skin with PASI scores (n = 23). Data represent the mean ± SEM. **P < 0.01, ***P < 0.001. Two-tailed unpaired Student’s t test was used.

**Figure 2:**
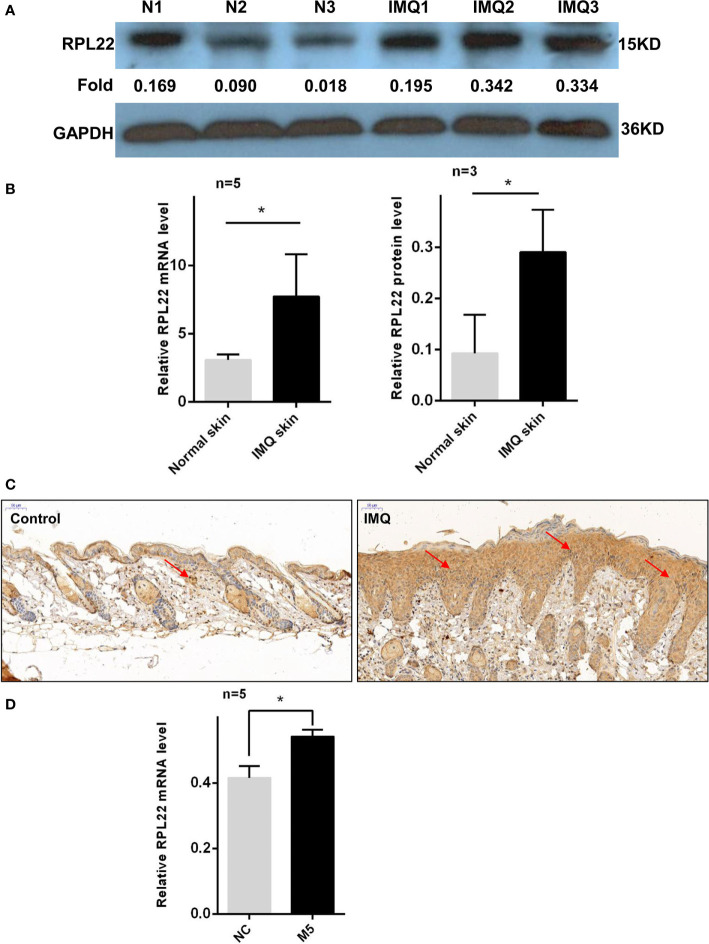
RPL22 expression is elevated in skin lesions of IMQ mouse models. **(A)** The protein expression level of RPL22 in lesional skins from IMQ-exposed mice and vehicle-exposed mice. **(B)** The statistical analysis of the relative mRNA (left panel, n = 5) and protein expression levels (right panel, n = 3) of RPL22 in lesional skins from IMQ-exposed mice and vehicle-exposed mice. **(C)** IHC staining of RPL22 in lesional skins from IMQ-exposed mice vehicle-exposed mice. **(D)** The mRNA expression level of RPL22 in psoriatic-like cells model on HaCaT cells stimulating with a cocktail of cytokines including Oncostatin-M, IL-1α, IL-17A, IL-22, and TNF-α(M5). Data **(D)** are representative of at least 3 independent experiments. Data represent the mean ± SEM. *P < 0.05. Two-tailed unpaired Student’s t test was used.

RT-qPCR and WB showed that RPL22 expression was upregulated in lesional skins from psoriasis patients compared with healthy controls (**Figures 1A, B**). In addition, we also established an IMQ-induced psoriasis mouse model, which closely resembled the human disease phenotype on BALB/c mice (17). Consistent with the results of human samples, mouse skins exposed to IMQ expressed significantly higher levels of RPL22 compared with vehicle-exposed mice (**Figures 2A, B**). IHC staining showed the increase of RPL22 expression occurred in both epidermis and dermis (**Figures 1C** and **2C**). Meanwhile, we found the mRNA expression level of RPL22 was significantly positive correlated with PASI score in psoriatic patients, which suggested RPL22 might act as a biomarker of disease severity (R=0.6813, n=23, P=0.0003) (**Figure 1D**).

Furthermore, we induced psoriatic-like cell model using HaCaT cells stimulated with a cocktail of cytokines including Oncostatin-M, IL-1α, IL-17A, IL-22, and TNF-α(M5) (15, 16, 18, 19). In cytokine-treated cells, qPCR showed that RPL22 expression was increased compared with control group (**Figure 2D**).

### RPL22 Regulates Cell Cycle *via* Controlling cyclinD1 Expression

**Figure 3:**
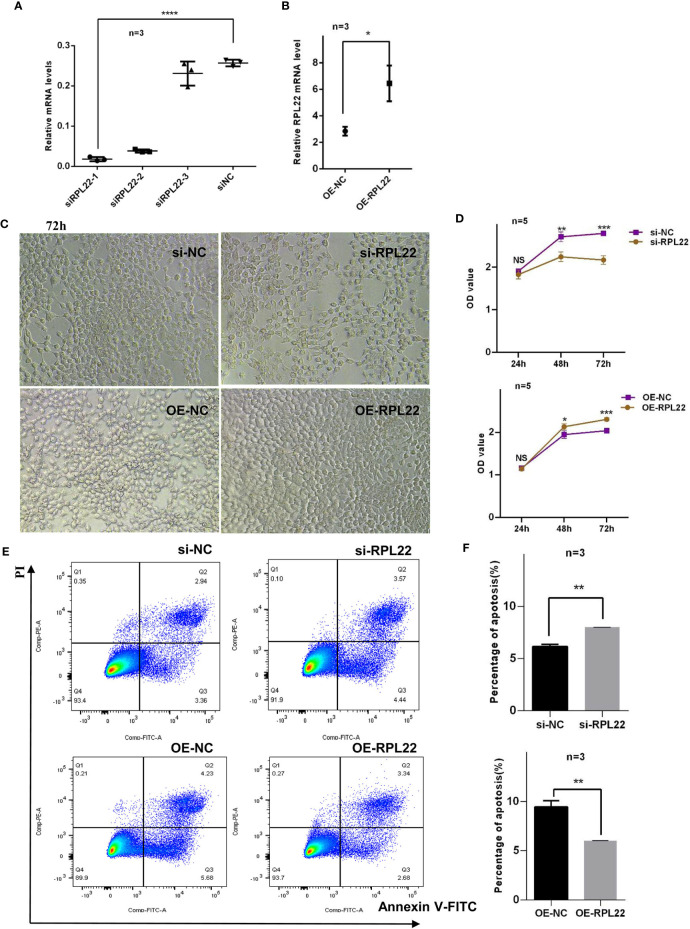
RPL22 promotes the proliferation of HaCaT cells and inhibits apoptosis of KCs. **(A)** Selection and verification of RPL22 siRNA intervention efficiency in HaCaT cells (n = 3). **(B)** Overexpression efficiency verification of RPL22 overexpressed plasmid (OE-RPL22) in HaCaT cells (n = 3). **(C)** Microscopy to observe cell proliferation state in HaCaT cells at 72hours. **(D)** CCK-8 assay to detect the cell proliferation level in HaCaT cells transfected with si-RPL22 or OE-RPL22 and their controls. **(E)** Flow cytometry to detect the apoptosis of KCs transfected with si-RPL22 or OE-RPL22 and their controls (n = 3). **(F)** Statistical analysis data to show the difference of four groups at 48 hours. All data are representative of at least 3 independent experiments. Data represent the mean ± SEM. *P < 0.05, **P < 0.01, ***P < 0.001, ****P < 0.0001. NS, not significant. Two-tailed unpaired Student’s t test was used.

**Figure 4:**
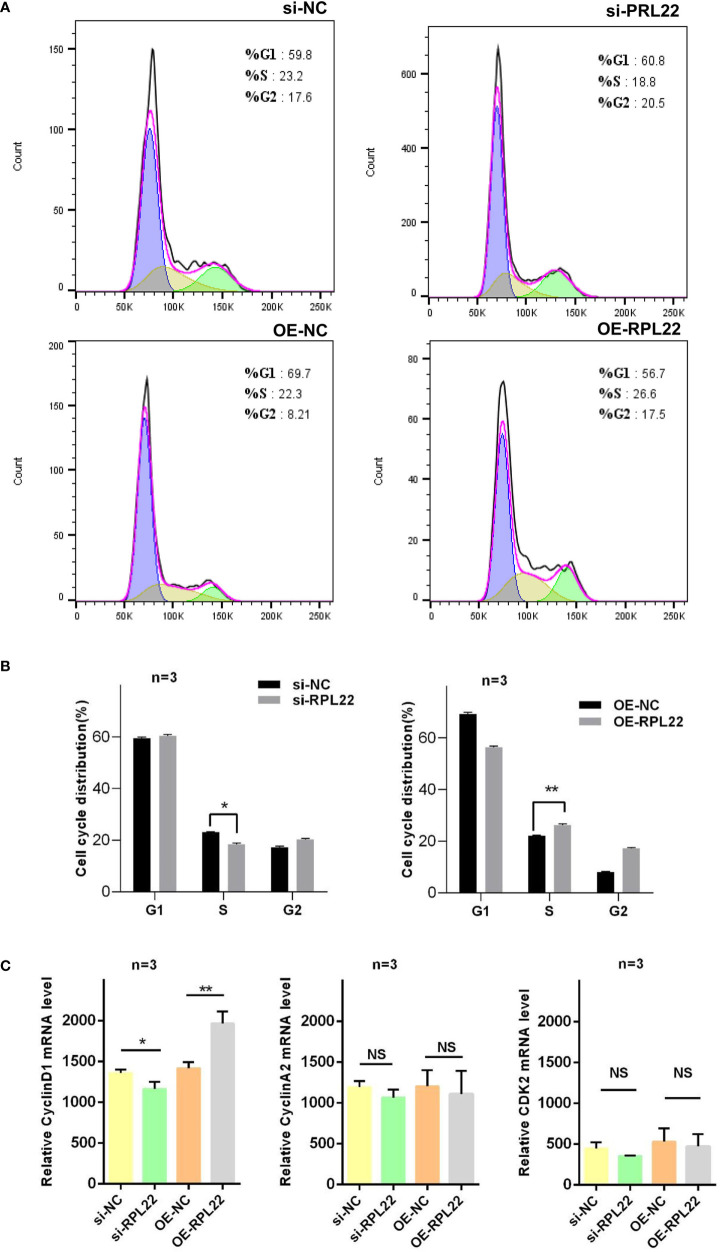
RPL22 is involved in the cell cycle. **(A)** Flow cytometry to detect the cell cycle of HaCaT cells transfected with si-RPL22 or OE-RPL22 and their controls (n = 3). **(B)** Statistical analysis data to show the difference of four groups at 48 hours. **(C)** The mRNA levels of cell cycle-related protein including CyclinD1, CyclinA2 and CDK2 in HaCaT cells transfected with si-RPL22 or OE-RPL22 and their controls (n = 3). All data are representative of at least 3 independent experiments. Data represent the mean ± SEM. *P < 0.05, **P < 0.01. NS, not significant. Two-tailed unpaired Student’s t test was used.

Abnormal proliferation and apoptosis of KCs are critical pathophysiological changes in PV (20). The proliferation progress of KCs in skin lesions of PV are accelerated, and the processes of apoptosis and differentiation are delayed (9). Since RPL22 is overexpressed in skin lesions of PV patients, we further investigated the role of RPL22 in KCs proliferation and apoptosis *in vitro*. First, we determined the most interference efficient siRNA(siRPL22-1, abbreviated as si-RPL22) to perform the following experiments (**Figure 3A**) and demonstrated the transfection efficiency of OE-RPL22 *in vitro *(**Figure 3B**). Next, CCK-8 assay indicated that the proliferation of HaCaT cells was inhibited by RPL22 knockdown and enhanced when RPL22 was upregulated by transfection with RPL22 overexpression plasmid at 48 and 72 hours compared with their control groups(**Figures 3C, D**). Furthermore, we carried out cellular apoptosis assay to examine the effect of RPL22 on KCs by flow cytometry. We found that RPL22 overexpression inhibited apoptosis and RPL22 knockdown promoted apoptosis (**Figures 3E, F**). To explore how RPL22 regulates proliferation, we carried out cell cycle analysis by flow cytometry and found that RPL22 knockdown decreased proportion of cells in S phase, whereas RPL22 overexpression had opposite effect (**Figures 4A, B**). We detected the expression levels of cell cycle-related protein such as cyclinA2, CDK2, CyclinD1 by RT-qPCR. CyclinD1 mRNA level was decreased in HaCaT cells transfected with si-RPL22 but was upregulated when RPL22 was overexpressed by plasmid. We didn’t observed alteration in the expression of cyclinA2 and CDK2 (**Figure 4C**). Together, these data suggested that RPL22 had a positive role in the proliferation of HaCaT cells and an anti-apoptotic role by targeting cyclinD1 to accelerate the cell cycle progression.

### RPL22 Enhances the Chemotaxis Ability of KCs by Inducing CXCL10 Expression and Promotes the Release of Psoriasis Related Inflammatory Cytokines *In Vitro*

**Figure 5:**
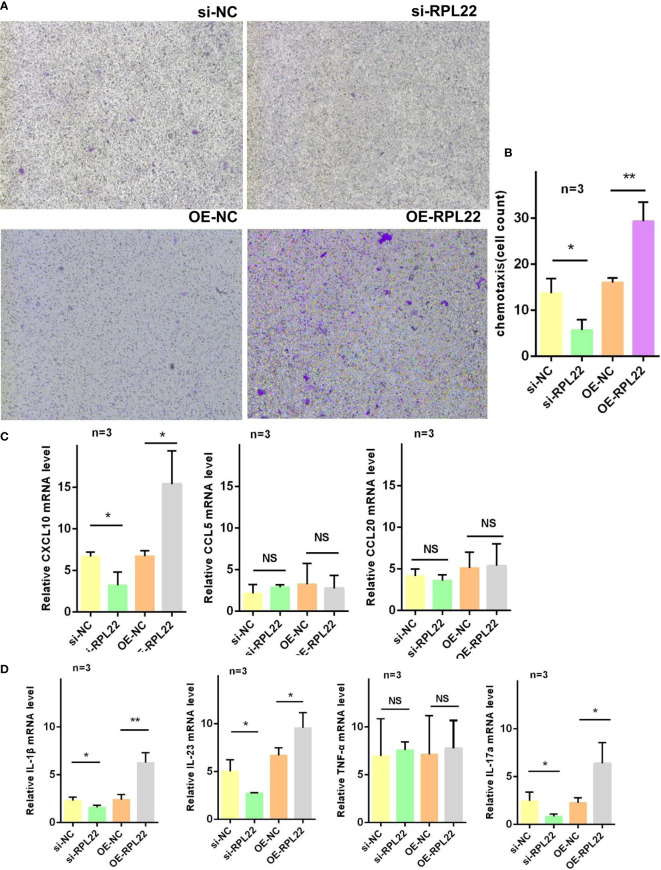
RPL22 promotes cell chemotaxis. **(A)** Chemotaxis assay to detect the chemotaxis efficiency of KCs transfected with si-RPL22 or OE-RPL22 and their controls on CD4^+^T cells (n = 3). **(B)** Cell count to statistically analyze the chemotaxis differences (n = 3). **(C)** RT-qPCR to detect the mRNA levels of chemokines of KCs transfected with si-RPL22 or OE-RPL22 and their controls including CXCL10, CCL5 and CCL20 (n=3). **(D)** RT-qPCR to detect the mRNA levels of psoriasis-associated cytokines of KCs transfected with si-RPL22 or OE-RPL22 and their controls including IL-1β, IL-23, TNF-α and IL-17a (n = 3). All data are representative of at least 3 independent experiments. Data represent the mean ± SEM. *P < 0.05, **P < 0.01. NS, not significant. Two-tailed unpaired Student’s t test was used.

The strengthened chemotaxis ability of KCs in PV is one of pathological manifestations. To demonstrate whether the upregulation of RPL22 plays a role in enhancing the chemotaxis ability of KCs, we co-cultured CD4^+^T cells from PBMCs of PV patients and primary KCs from foreskin. We observed that RPL22 overexpression promoted CD4^+^T cells chemotaxis to KCs and RPL22 knockdown showed the opposite results (**Figures 5A, B**). Correspondingly, we determined the mRNA expression levels of chemokines including CXCL10, CCL5 and CCL20. The level of CXCL10 expression was increased by RPL22 overexpression and consistently, RPL22 knockdown showed the opposite result. The levels of CCL5 and CCL20 were unaffected. This suggested that RPL22 enhanced the chemotaxis ability of KCs by inducing CXCL10 expression (**Figure 5C**).

Similarly, our studies also detected the expression of psoriasis-associated cytokines such as IL-1β, IL-23, TNF-α and IL-17a by RT-qPCR and found the levels of IL-1β, IL-23 and IL-17a were significantly increased while TNF-α showed no change in RPL22-overexpressed KCs, which was in accordance with RPL22 knockdown (**Figure 5D**). These results suggested that RPL22enhanced the chemotaxis ability of KCs by inducing CXCL10 expression and promoted the release of psoriasis related inflammatory cytokines *in vitro.*

### H3K27ac Modification in Promoter Region of RPL22 Increases Its Expression

**Figure 6:**
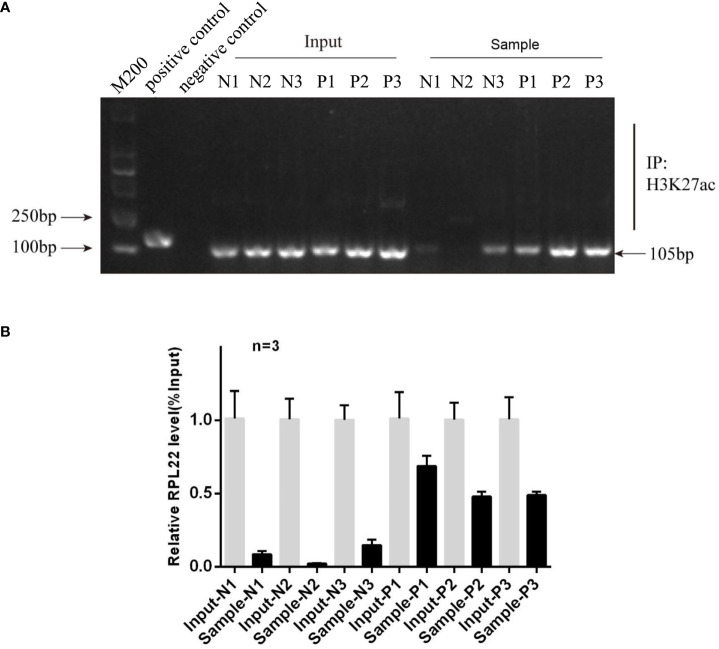
The hyperacetylation level in the promoter region of RPL22 elevates its expression. **(A)** Electrophoretic image of ChIP assay result showed the histone H3K27 acetylation level on RPL22 promoter in skin lesions derived from PV patients was significantly higher than healthy controls (n = 3). **(B)** ChIP-qPCR showed the enrichment of H3K27ac in RPL22 promoter in all three skin lesions derived from PV patients was significantly higher than healthy controls.

To further investigate the mechanisms of increasing RPL22 expression in the skin lesion of psoriatic patients, we used UCSC Genome Browser software to predict the acetylation activity in the enhancer of histone H3K27 in RPL22 promoter region. We conducted a ChIP-qPCR assay and compared the histone H3K27 acetylation (H3K27ac) on RPL22 promoter in skin lesions of PV patients and healthy skin controls. Markedly, psoriatic skins exhibited increased H3K27ac level on RPL22 promoter compared with normal skins (**Figures 6A, B**). These data indicated that the increased expression of RPL22 in PV patients was attributed to the hyperacetylation of H3K27 on RPL22 promoter.

### RPL22 Overexpression Accelerates the Development of Psoriatic Lesions

**Figure 7:**
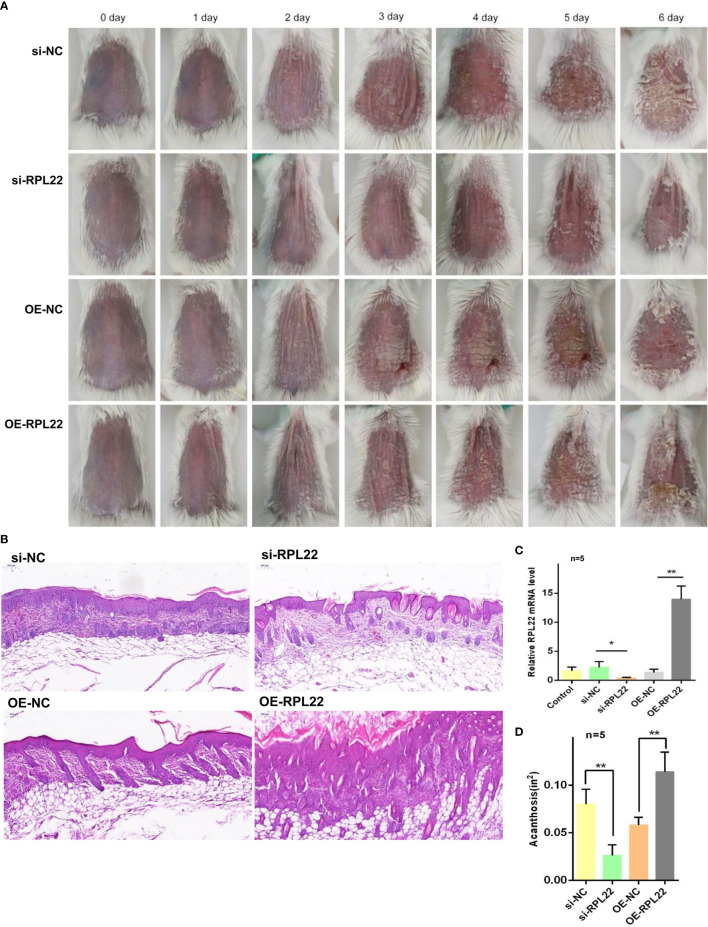
RPL22 overexpression accelerates the development of psoriatic lesions **(A)** Clinical efficacies on skin lesions in IMQ mice models after RPL22 intervention in four groups of si-RPL22, si-NC, OE-RPL22 and OE-NC. **(B)** H&E staining of lesional skin from mice in the above four groups to show pathological manifestation including acanthosis and dermal inflammatory cells infiltration. **(C)** RT-qPCR to detect the RPL22 mRNA expression level in lesional skin from IMQ-induced psoriasis-like mouse model intradermally injected with si-RPL22 or si-NC or OE-RPL22 or OE-NC (n = 5). **(D)** Acanthosis was quantified for IMQ-induced mice treated with si-RPL22 or si-NC or OE-RPL22 or OE-NC (n = 5). Data represent the mean ± SEM. *P < 0.05, **P < 0.01. Two-tailed unpaired Student’s t test was used.

To explore the critical role of RPL22 upregulation in the development of psoriasis *in vivo*, we established IMQ induced psoriasis-like mouse model and simultaneously injected with OE-RPL22 or si-RPL22 intradermally daily for the first three consecutive days (0/1/2/3 day). RT-qPCR demonstrated that the overexpression or intervention efficiency of RPL22 expression (**Figure 7C**). Compared with control group, the overexpression of RPL22 showed accelerated progresses and increased disease severity, which was most significant on the sixth day (**Figures 7A, D**). Acanthosis and dermal inflammatory cell infiltration were also significantly increased after RPL22 overexpression (**Figure 7B**). Consistently, the si-RPL22 group showed visible improvement in both clinical and pathological manifestation including acanthosis and dermal inflammatory cell infiltration compared with control (**Figures 7A, B, D**). These results demonstrated the pathological function of RPL22 in psoriasis and decreased-expression of RPL22 was expected to inhibit the development of psoriasis.

## Discussion

Ribosomal protein (RP) is a ribosome binding protein that participates in the biological formation of ribosomes and protein synthesis. Recently, it has been discovered that RP can also bind to specific mRNA and play a regulatory role. Previous studies have found that some free RP, for example, RPL5, RPL11, can affect cell cycle and proliferation by regulating P53 pathway (21). RPL22 has also recently been reported to involve in cell proliferation and apoptosis regulation (22, 23). Current studies referring to RPL22 mainly focus on tumor studies, such as T cells lymphoma (14),colon cancer (24), adrenal cortical carcinoma, etc. (25), but have not been reported in inflammatory skin diseases such as psoriasis. Therefore, this is the first study to report the differential expression and role of RPL22 in inflammatory skin diseases. In this study, we found that the RPL22 expression in the skin lesions of psoriasis patients and IMQ-induced psoriasis-like mouse models well as psoriasis-like HaCaT cells was significantly increased, and RPL22 could also promote keratinocytes proliferation, inhibit keratinocytes apoptosis and induce inflammatory cell chemotaxis *in vitro*, further aggravate the progression of psoriasis. Inhibiting the expression of RPL22 can improve the clinical and pathological manifestations of IMQ-induced psoriasis mouse models, and overexpression of RPL22 can significantly promote psoriasis progression.

We found overexpression of RPL22 elevated the proportion of cells entering S phase which suggested RPL22 enhanced keratinocytes proliferation. After intervention with the expression of RPL22, we also detected the expression of cell cycle-related proteins in HaCaT cells including CyclinA2, CDK2 and Cyclin D1, and found that the cyclinD1 level was significantly changed, while CDK2 and cyclinA2 did not change obviously, which indicated that RPL22 might promote keratinocytes proliferation and inhibit their apoptosis by targeting cyclinD1. CyclinD1 is a cell cycle regulator and forms a complex with CDK4 (26, 27). CyclinD1-CDK4 can phosphorylate retinoblastoma gene(RB), which leads to E2F transcription factors release and G1-S cell cycle conversion, further shorten the cell cycle, and promote cell proliferation (28, 29). Although previous studies reported that RPL22 might act as a protective factor to promote cell senescence under physiological conditions (23), while in pathological conditions such as inflammation were caused by immune disorders. RPL22 might be overexpressed under the control of other pathways, which might lead to different downstream pathways, while further studies are required to confirm the hypothesis. Here, we believe that RPL22 plays a destructive role in psoriasis. It facilitates the pathogenesis by promoting the keratinocyte proliferation, inflammatory cell chemotaxis and inflammation. This discrepancy in the effects of RPL22 on cell proliferation may be attributed to the body’s immune status and the components of the lesion microenvironment. The occurrence of tumors is related to immune disorders. The body’s immune functions of recognizing and eliminating tumor cells is insufficient, especially the CD4^+^T cells-mediated humoral immunity and CD8^+^ T cell-mediated cytotoxicity, which make abnormal cells proliferate and differentiate out of control. Inflammatory diseases including psoriasis are manifested as an overreaction of inflammation and its skin lesions are enriched with CD4^+^ T cells such as Th17 and high levels of inflammatory factors. This inflammatory environment in the skin may change the function of RPL22 by affecting the downstream targets of RPL22. In this study, we believe that the inflammatory factors in the microenvironment affect the promotion of RPL22 on cyclinD1, which leads to over proliferation of KCs.

Chemokines and inflammatory factors are essential in the inflammatory response in psoriasis. Previous reports have shown that CXCL10 expression increases in the plasma of psoriasis patients and may be a significant factor in psoriatic arthritis development (30). CXCL10 is an essential member for chemotaxis of CD4^+^ T cells, eosinophils, monocytes and other leukocytes to the skin lesions (31). In addition, IL-1β, IL-23 and IL-17a are also very important cytokines involved in the pathogenesis of psoriasis. In our study, overexpression of RPL22 could promote CD4^+^T chemotaxis to KCs and increased the expression of CXCL10, IL-1β, IL-23 and IL-17a. Therefore, we supposed RPL22 might target CXCL10 to promote chemotaxis of inflammatory cells and aggravate local inflammatory response. In addition, our results show that RPL22 affects cytokines production in KCs. KCs play critical roles in pathogenesis of psoriasis by interacting with immune cells and secreting cytokines and chemokines. Thus, we hypothesize that overexpression of RPL22 reduces pro-inflammatory molecules production in epidermis and thus strengthens the interaction between KCs and immune cells. Our *in vitro* data also suggests that knockdown of RPL22 has a direct inhibitory effect on KCs proliferation, which may result in the thinner epidermis in siRPL22 injected mouse skins.

Studies have confirmed that psoriasis is a polygenic genetic disease induced by environmental factors. Epigenetic modification, as an important bridge for environmental factors to affect the disease occurrence and development, has gradually attracted attention. These regulatory mechanisms mainly include DNA methylation, histone modification, and non-coding RNA regulation. In order to further explain the specific mechanism of the increased expression of RPL22 in psoriasis lesions, we predicted that there were H3K27 acetylated islands in the upstream promoter region of RPL22 through the UCSC Genome Browser (32), suggesting that this region might have active enhancers. Furtherly, we used ChIP-qPCR technology to detect the histone H3K27 acetylation level in the RPL22 promoter region in psoriasis lesions and found there were indeed hyperacetylated modification in the upstream of RPL22 sequence. But the exact regulation mechanisms of the acetylation level in the RPL22 promoter region in psoriasis lesions is unclear, which requires further study. Moreover, there are a few limitations in this study: 1) we did not use the RPL22 transgenic mice models or KO animal models on genetic animal models of psoriasis to directly claim the mechanism of increased RPL22 expression to involve in the occurrence and development of psoriasis; 2) In this study, we have not yet completed the relevant detection of cell differentiation markers, and whether RPL22 has an effect on cell differentiation needs further confirmation in subsequent experiments; 3) In the mice models, we paid more attention to phenotypic changes, and have not yet further improved the detection of related inflammatory factors.

**Figure 8:**
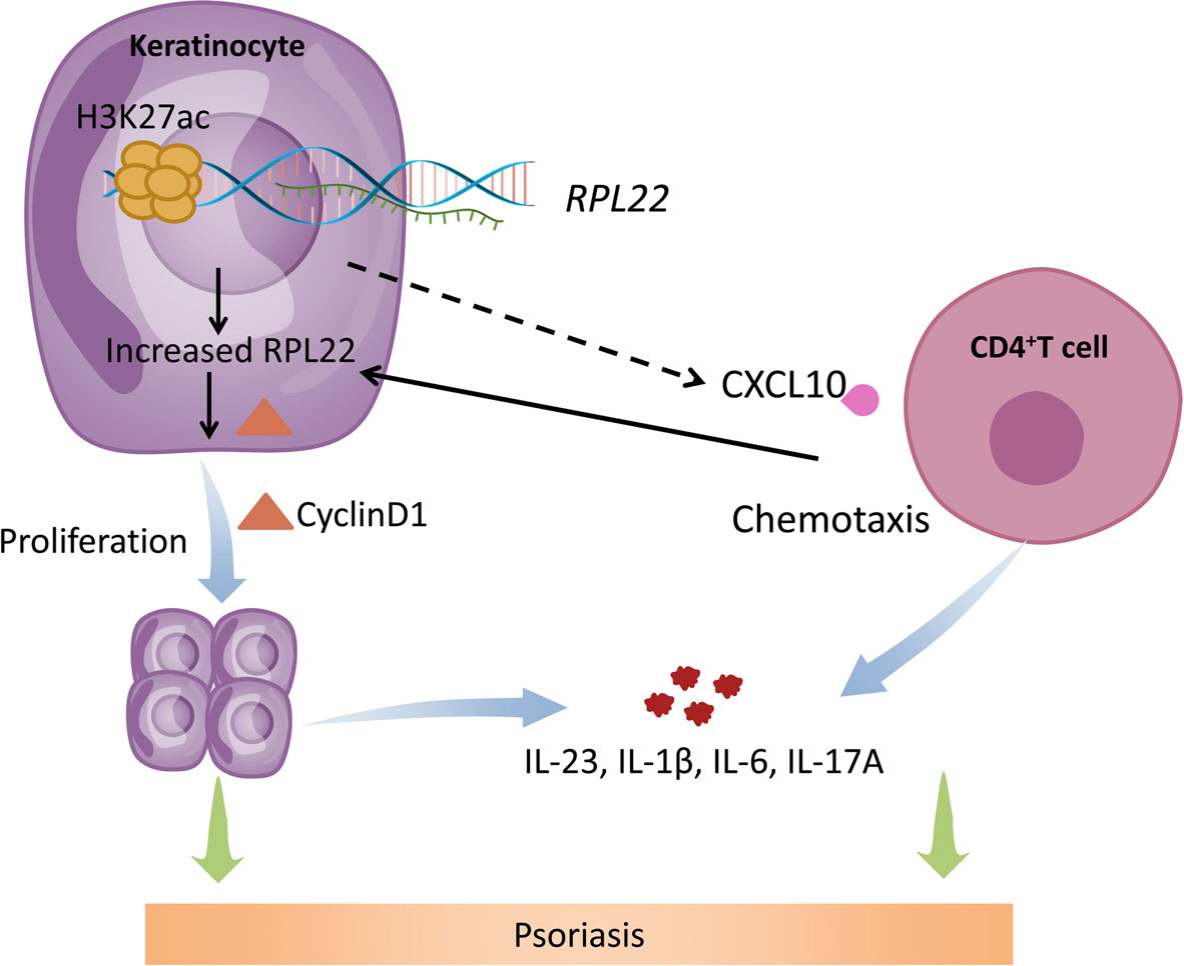
Schematic illustration of RPL22 contributing to the pathogenesis of psoriasis. In psoriasis patients, hyperacetylation of histone H3K27 in the promoter region of RPL22 in the skin lesions of psoriasis patients induces RPL22 overexpression, overexpressed RPL22 up-regulates CyclinD1 expression to promote the KCs proliferation and enhancing CXCL10 expression to induce CD4^+^ T cells chemotaxis, Both of them secrete cytokines including IL-23, IL-6, IL-1β and IL-17a to aggravate the occurrence and development of psoriasis.

In summary, our study revealed that hyperacetylation of histone H3K27 in the promoter region of RPL22 in the skin lesions of psoriasis patients induced RPL22 overexpression, upregulated RPL22 controlled the cell cycle by up-regulating the expression of cyclinD1 to promote the keratinocytes proliferation and enhanced the chemotaxis of CD4^+^ T cells by up-regulating CXCL10 expression in KCs to induce inflammatory reaction to participate in the occurrence and development of psoriasis (**Figure 8**). Therefore, RPL22 can aggravate the progression of psoriasis by inducing abnormal biological behaviors of keratinocytes which is expected to become a novel therapeutic target in the future.

## Data Availability Statement

The original contributions presented in the study are included in the article/**Supplementary Material**. Further inquiries can be directed to the corresponding authors.

## Ethics Statement

The studies involving human participants were reviewed and approved by The IRB Third Xiangya Hospital, Central South University. The patients/participants provided their written informed consent to participate in this study. The animal study was reviewed and approved by The IRB of Third Xiangya Hospital, Central South University. All the biosafety measurements have been adopted and the institutional safety procedures are adhered. The laboratory of our institution has biosafety level 1 (BSL-1) standard where all standards and protocols are adopted as per the guidelines of CLSI.

## Author Contributions

JZ performed the experiments and drafted the manuscript. JL conceived and designed the study. SD, YZ, and HZ analyzed the data. YZZ, QH, YX, and CC performed parts of experiments and formal analysis. XT and LG reviewed and edited the manuscript. All authors contributed to the article and approved the submitted version.

## Funding

This work was supported by the New Xiangya Talent Projects of the Third Xiangya Hospital of Central South University (Grant No. 20170309).

## Conflict of Interest

The authors declare that the research was conducted in the absence of any commercial or financial relationships that could be construed as a potential conflict of interest.
