# Supplementary figures and images for: RPL22 Overexpression Promotes Psoriasis-Like Lesion by Inducing Keratinocytes Abnormal Biological Behavior
Source: Front Immunol. 2021 Jun 18;12:699900. doi: 10.3389/fimmu.2021.699900 (PMC8250439; doi:10.3389/fimmu.2021.699900)

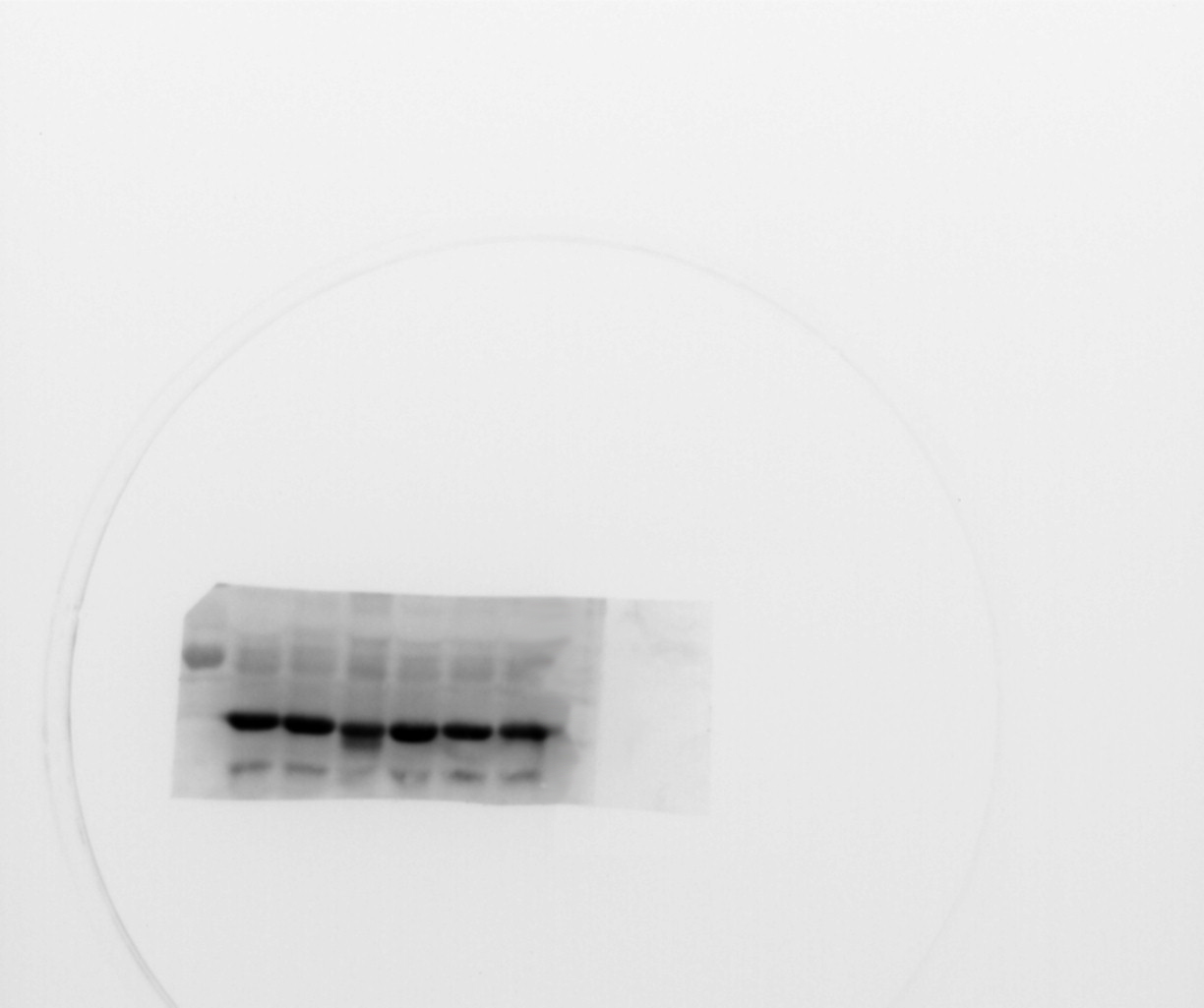

Supplement: Supplementary file 1 [file Image_1.jpeg]

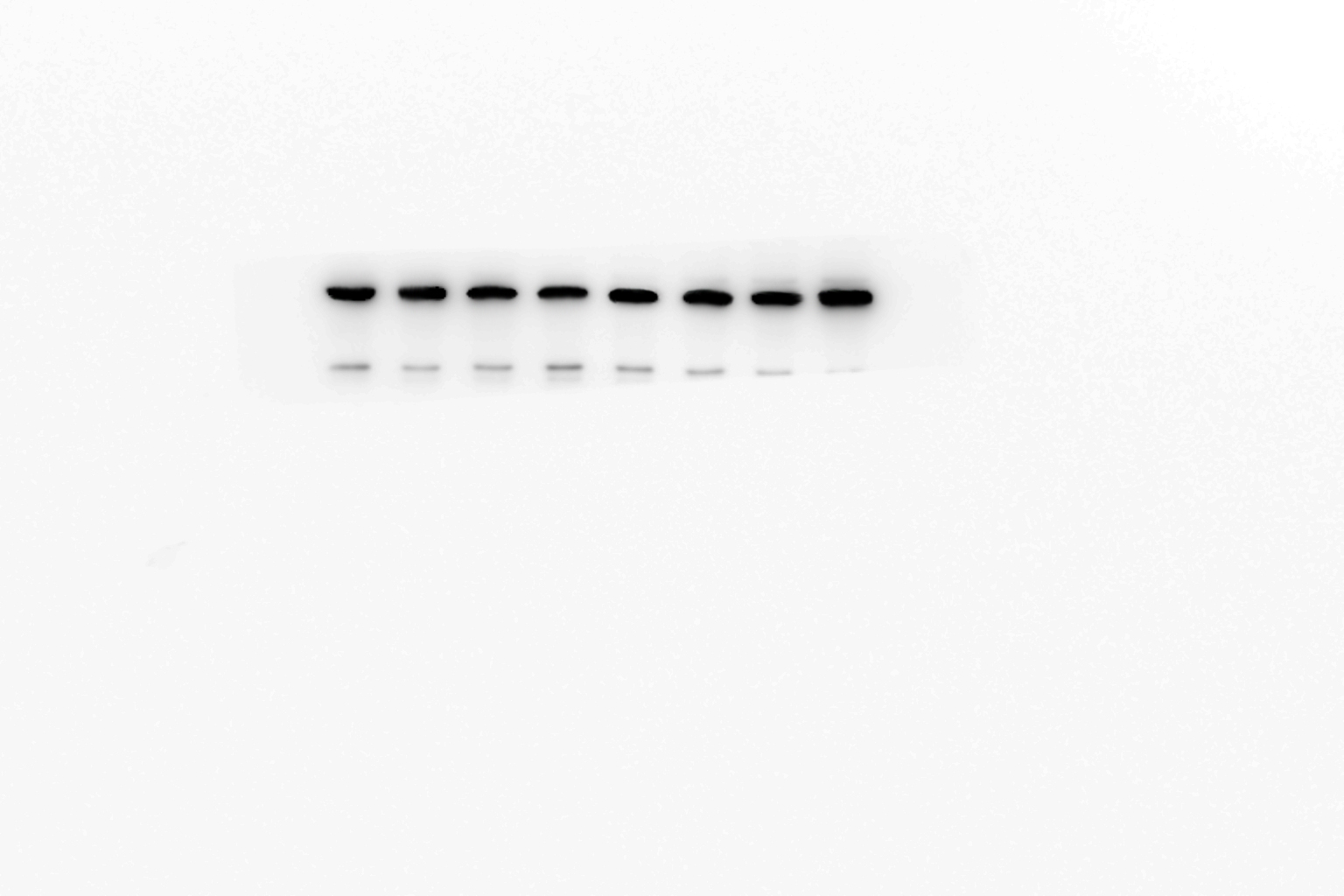

Supplement: Supplementary file 2 [file Image_2.jpeg]

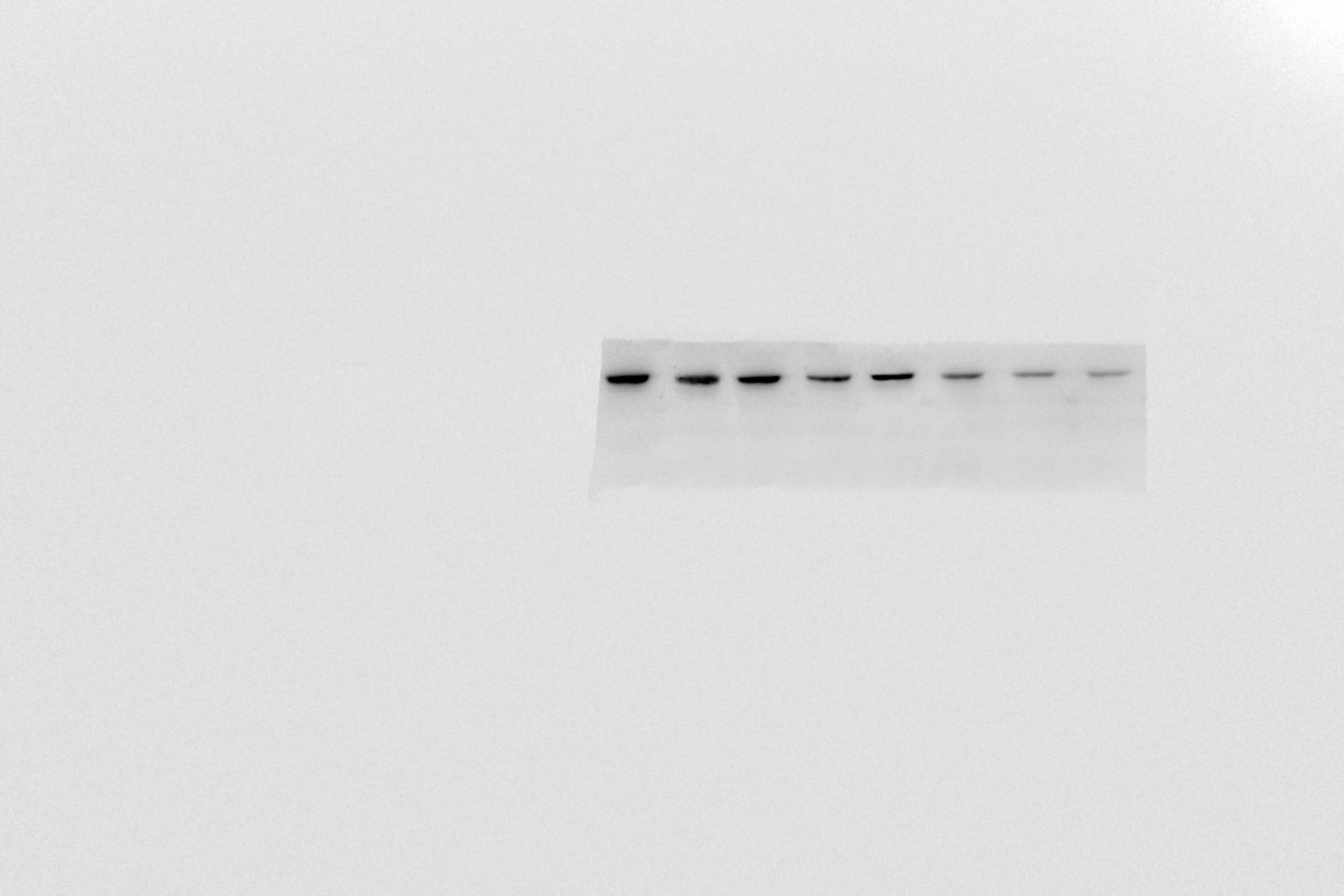

Supplement: Supplementary file 4 [file Image_4.jpeg]

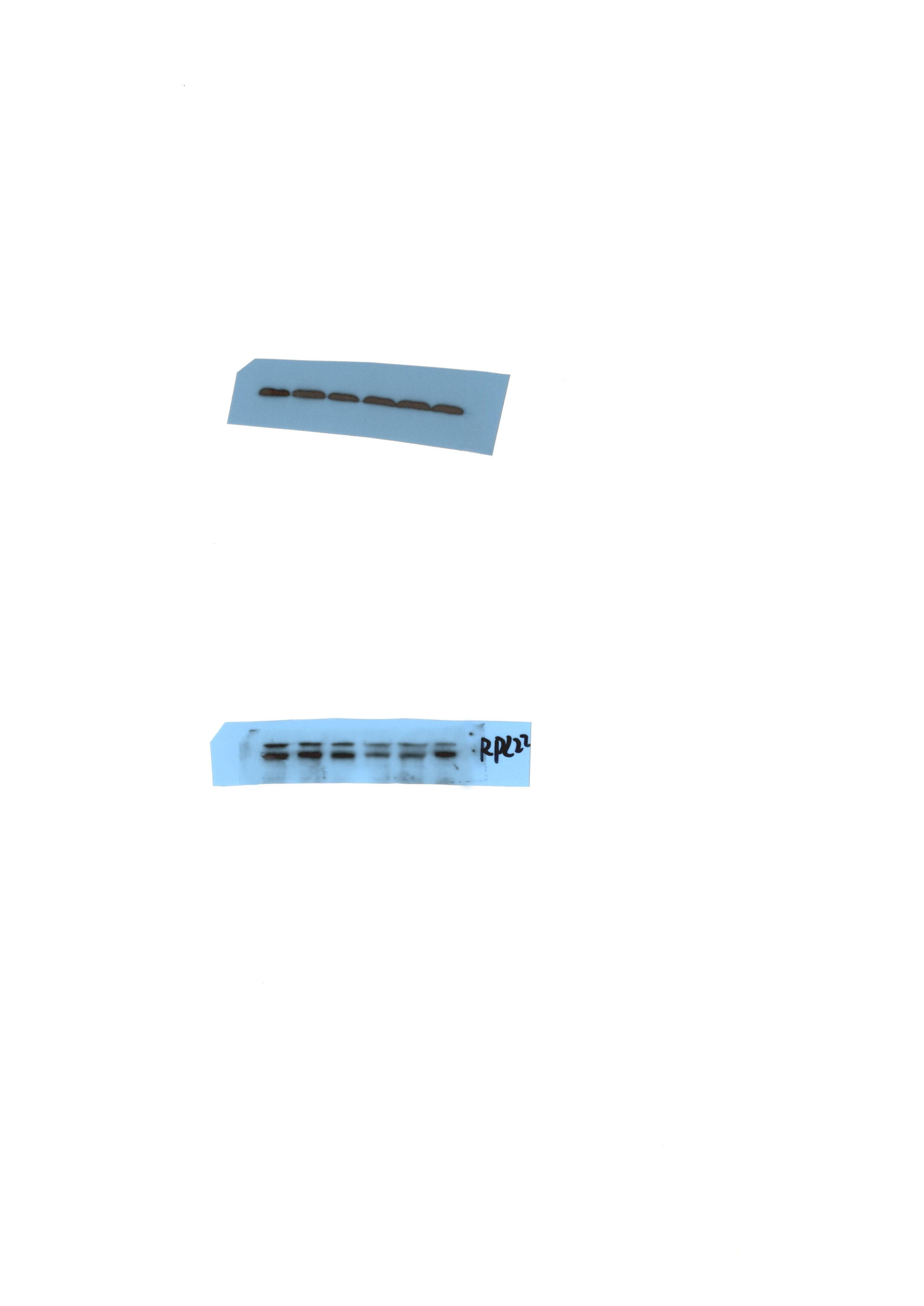

Supplement: Supplementary file 5 [file Image_5.jpeg]

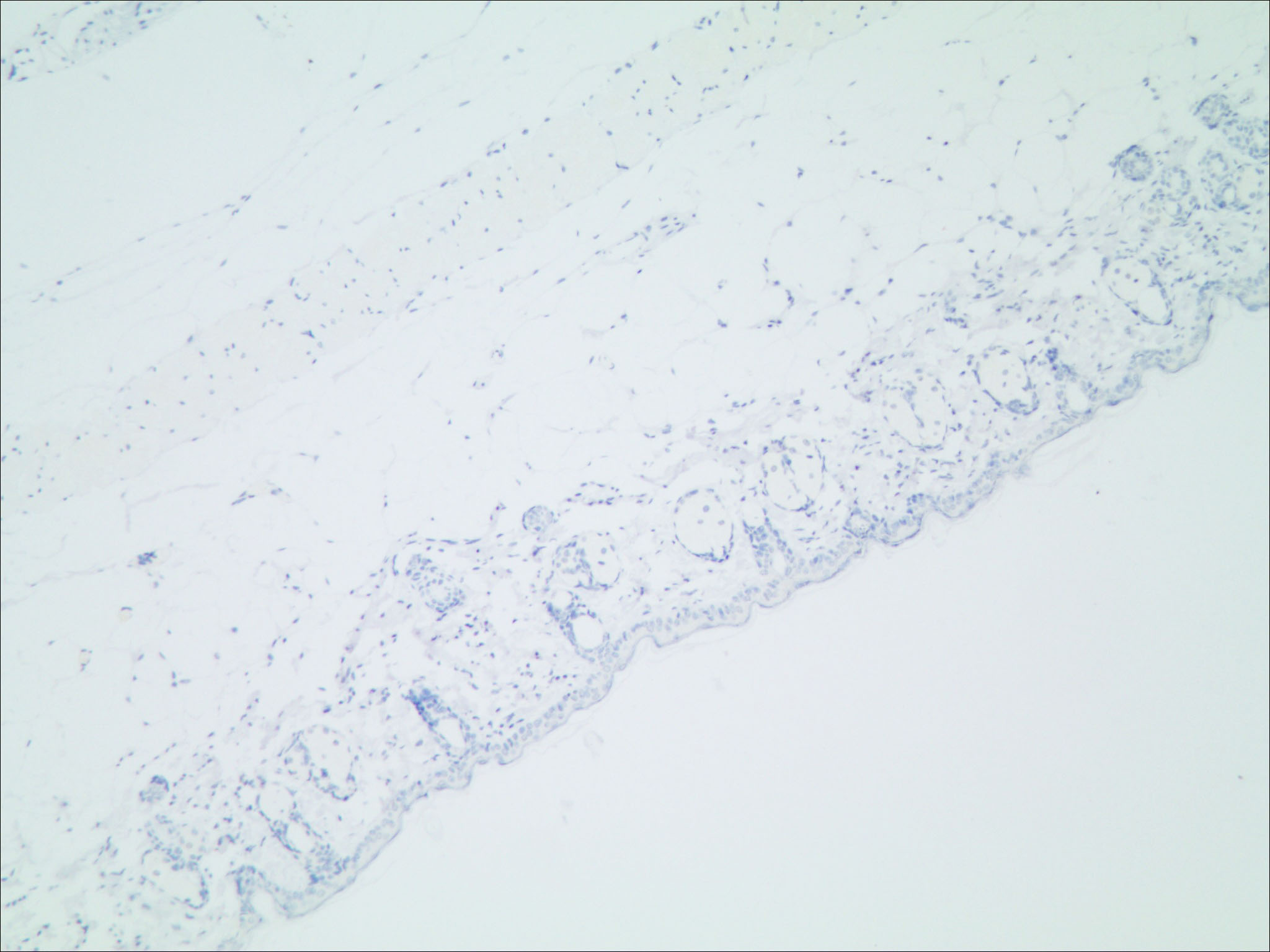

Supplement: Supplementary file 6 [file Image_6.jpeg]

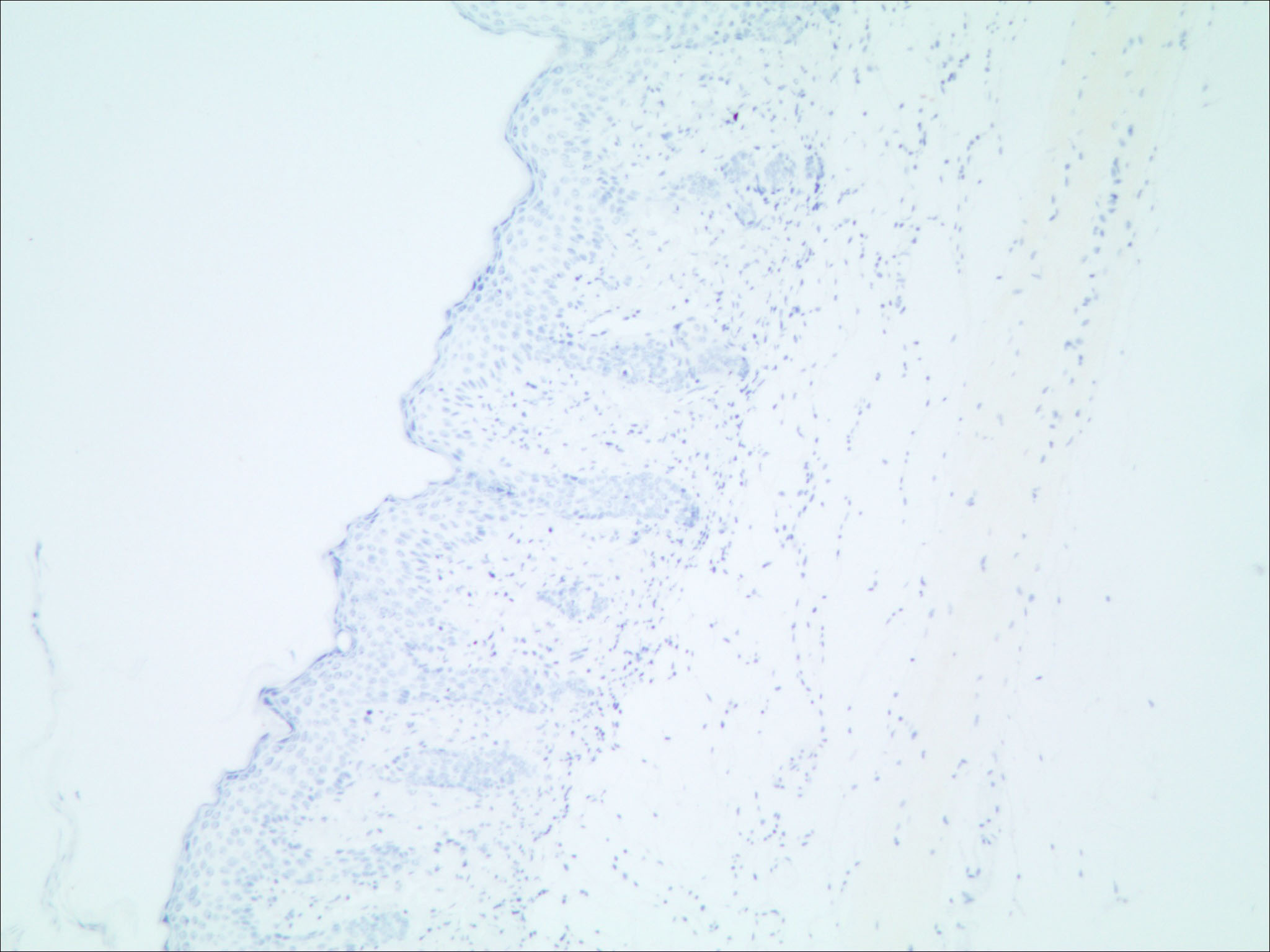

Supplement: Supplementary file 7 [file Image_7.jpeg]

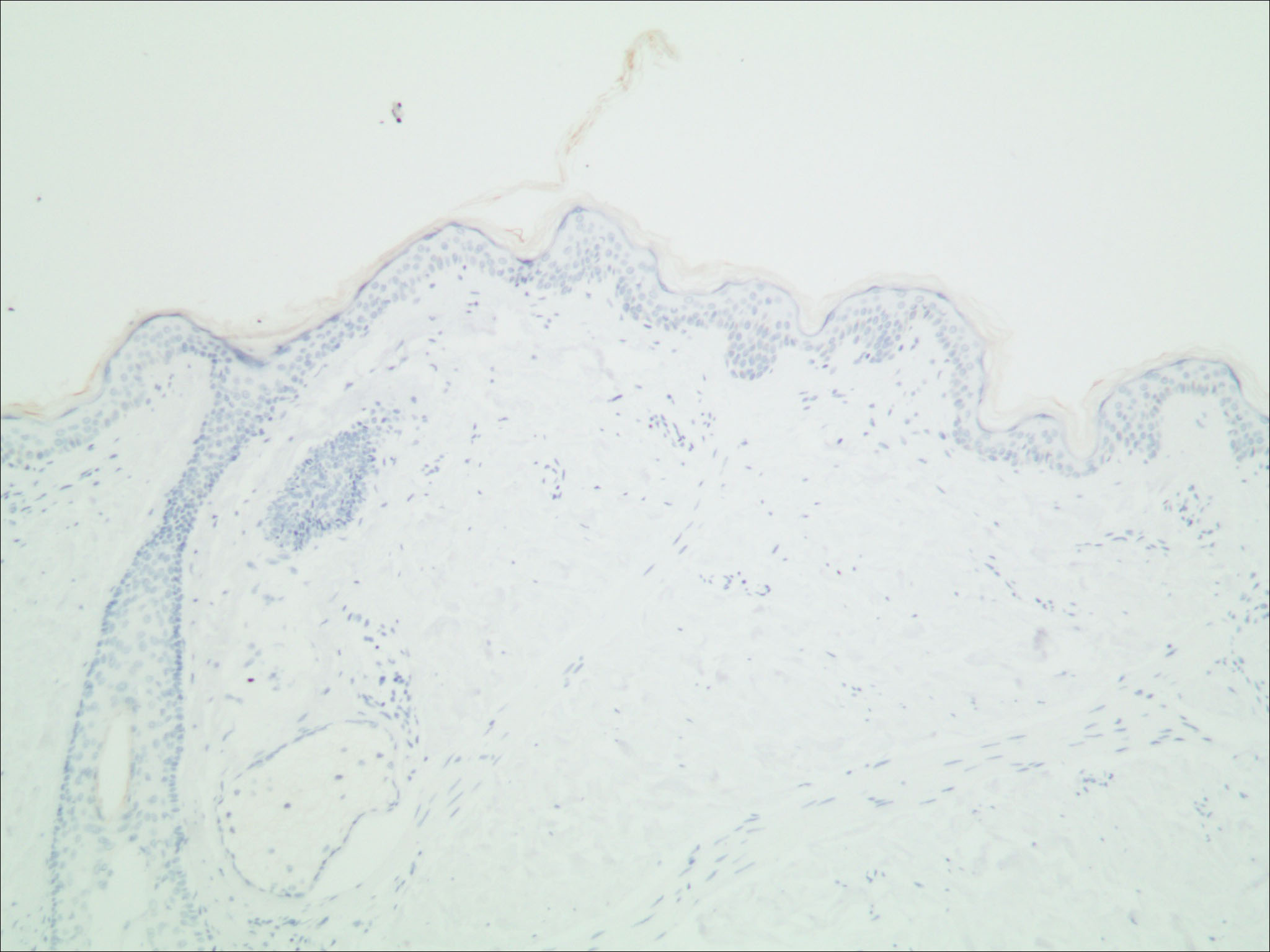

Supplement: Supplementary file 8 [file Image_8.jpeg]

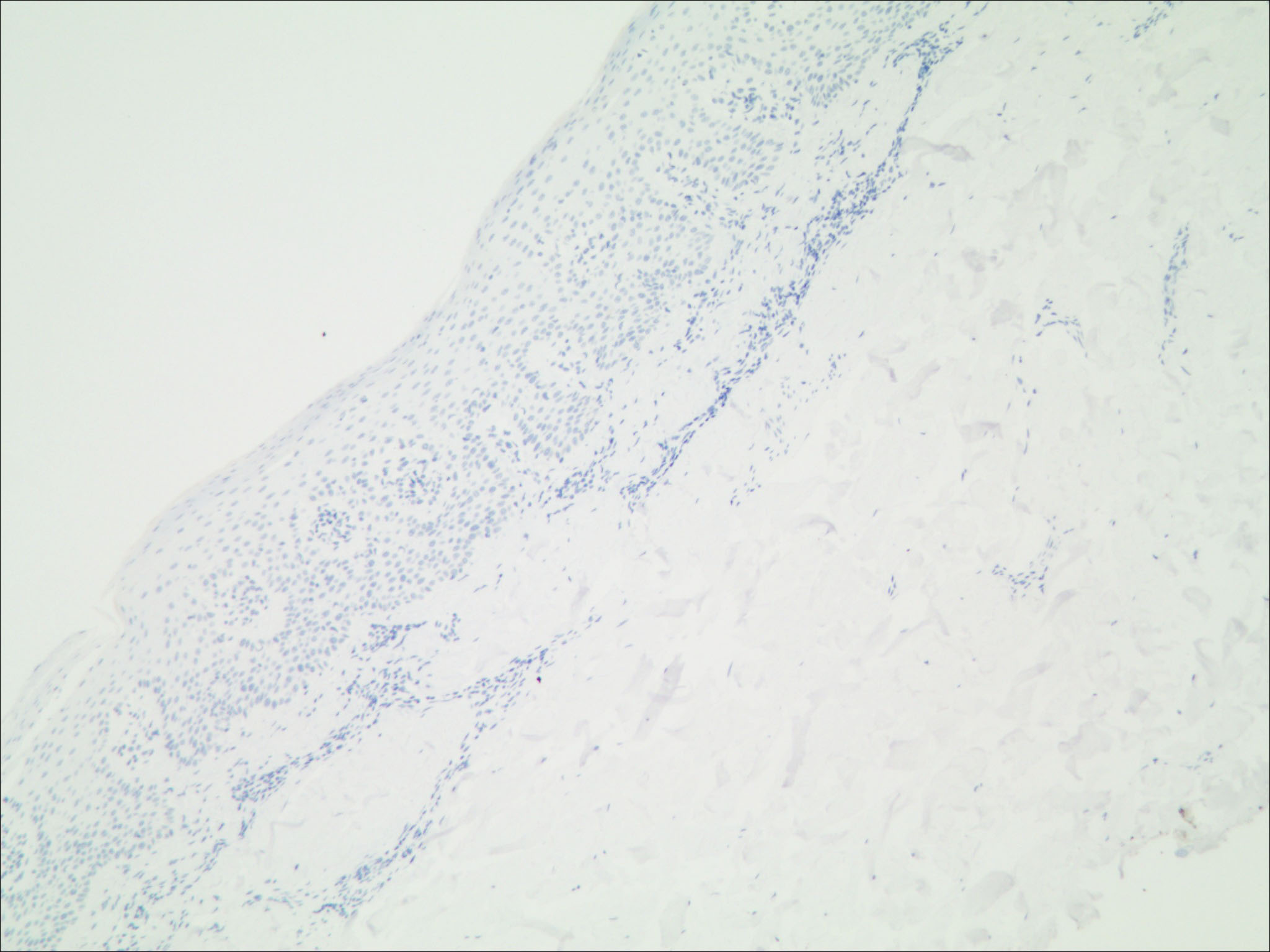

Supplement: Supplementary file 9 [file Image_9.jpeg]
