# Supplementary material for: RPL22 Overexpression Promotes Psoriasis-Like Lesion by Inducing Keratinocytes Abnormal Biological Behavior
Source: Front Immunol. 2021 Jun 18;12:699900. doi: 10.3389/fimmu.2021.699900 (PMC8250439; doi:10.3389/fimmu.2021.699900)
Supplement: Supplementary file 10 [file Table_1.docx]

**Supplementary table 1. Clinical information of enrollees**

| Sample ID | Age/Gender | PASI score |
| --- | --- | --- |
| 1 | 31/M | 17.5 |
| 2 | 41/M | 20.8 |
| 3 | 39/F | 15.6 |
| 4 | 55/M | 30.1 |
| 5 | 21/M | 10.6 |
| 6 | 41/M | 24.2 |
| 7 | 25/M | 18.9 |
| 8 | 41/M | 7.2 |
| 9 | 51/F | 18.8 |
| 10 | 36/F | 18.6 |
| 11 | 48/F | 29.2 |
| 12 | 37/M | 14.5 |
| 13 | 45/F | 23.0 |
| 14 | 56/F | 10.6 |
| 15 | 27/F | 20.6 |
| 16 | 59/M | 15.2 |
| 17 | 25/F | 7.8 |
| 18 | 68/M | 16.7 |
| 19 | 54/M | 8.2 |
| 20 | 24/M | 18.0 |
| 21 | 18/M | 10.9 |
| 22 | 54/M | 23.9 |
| 23 | 33/F | 25.4 |
