# Supplementary material for: RPL22 Overexpression Promotes Psoriasis-Like Lesion by Inducing Keratinocytes Abnormal Biological Behavior
Source: Front Immunol. 2021 Jun 18;12:699900. doi: 10.3389/fimmu.2021.699900 (PMC8250439; doi:10.3389/fimmu.2021.699900)
Supplement: Supplementary file 11 [file Table_2.docx]

**Supplementary table 2. Primer sequences in the article**

| **Quantitative PCR primers** | |
| --- | --- |
| RPL22(H) | F:5’-CTGCCAATTTTGAGCAGTTCCTCCA-3’  R:5’-TCAAATACCTTTTGGAGAAAGGCACC-3’ |
| GAPDH(H) | F:5’-GGAGCGAGATCCCTCCAAAAT-3’  R:5’-GGCTGTTGTCATACTTCTCATGG-3’ |
| CyclinD1(H) | F:5’-GTCCTACTTCAAATGTGTGCAG-3’  R:5’-GGGATGGTCTCCTTCATCTTAG-3’ |
| CyclinA2(H) | F:5’-AGAAACAGCCAGACATCACTAA-3’  R:5’-TTCAAACTTTGAGGCTAACAGC-3’ |
| CDK2(H) | F:5’-CCTGGGCTGCAAATATTATTCC-3’  R:5’-TGGCTTGTAATCAGGCATAGAA-3’ |
| CXCL10(H) | F:5’-GGTGAGAAGAGATGTCTGAATCC-3’  R:5’-GTCCATCCTTGGAAGCACTGCA-3’ |
| CCL5(H) | F:5’-CCTGCTGCTTTGCCTACATTGC-3’  R:5’-ACACACTTGGCGGTTCTTTCGG-3’ |
| CCL20(H) | F:5’-AAGTTGTCTGTGTGCGCAAATCC-3’  R:5’-CCATTCCAGAAAAGCCACAGTTTT-3’ |
| IL-1β(H) | F:5’-CCACAGACCTTCCAGGAGAATG-3’  R:5’-GTGCAGTTCAGTGATCGTACAGG-3’ |
| TNF-α(H) | F:5’-GGACACCATGAGCACTGAAAGC-3’  R:5’-TGCCACGATCAGGAAGGAGAAG-3’ |
| IL-23(H) | F:5’-GAGCCTTCTCTGCTCCCTGATA-3’  R:5’-GACTGAGGCTTGGAATCTGCTG-3’ |
| IL-17a(H) | F:5’-ATTACTACAACCGATCCACCTC-3’  R:5’-TGGTAGTCCACGTTCCCAT-3’ |
| RPL22(M) | F:5’-TGTGCTCTCTCTGCAGGTAT-3’  R:5’-CCAGTCTCGGAGGTTGTTCT-3’ |
| GAPDH(M) | F:5’-AGGTCGGTGTGAACGGATTTG-3’  R:5’-TGTAGACCATGTAGTTGAGGTCA-3’ |
| **ChIP-qPCR primers** | |
| RPL22(H) | F:5’-AACAGAGCGAGAGCCTGTCT-3’ |
|  | R:5’-CAGCCCCTTCTTCCTAAACC-3’ |
